# The Association of Emotion Regulation and Somatic Symptoms

**DOI:** 10.1097/PSY.0000000000001310

**Published:** 2024-04-11

**Authors:** Tara M. Petzke, Michael Witthöft

**Affiliations:** From the Department of Clinical Psychology, Psychotherapy, and Experimental Psychopathology, Johannes-Gutenberg-University Mainz, Mainz, Germany.

**Keywords:** persistent somatic symptoms, functional disorders, emotions, emotion regulation, **ANOVA** = analysis of variance, **ER** = emotion regulation, **ERQ** = Emotion Regulation Questionnaire, **HiTOP** = Hierarchical Taxonomy of Psychopathology, **PHQ** = Patient Health Questionnaire, **SEM** = structural equation model, **SSD** = somatic symptom disorder

## Abstract

**Objective:**

People with functional somatic symptoms have difficulties in various stages of the emotion regulation (ER) process. As an adaptive and flexible use of ER strategies is a core tenet of emotional health, having difficulties in this area is often assumed to be the key mechanism behind functional somatic symptoms. Following a dimensional population-based sampling approach, we investigated ER abilities across a broad range of people and tested possible associations with somatic symptom reporting, habitual ER use, and various subclinical constructs (such as alexithymia and anxiety).

**Methods:**

In a sample of *N* = 254 persons, somatic symptom distress (Patient Health Questionnaire-15, Hierarchical Taxonomy of Psychopathology somatoform spectrum), trait ER facets (Emotion Regulation Questionnaire, Emotion Reactivity Scale), and the ER abilities (suppression and reappraisal) were assessed. Correlations (frequentist and Bayesian), ANOVAs, and structural equation models were used to analyze the data.

**Results:**

Correlational and structural equation model analyses revealed that general symptom severity (both on the somatoform Hierarchical Taxonomy of Psychopathology and Patient Health Questionnaire-15) was not significantly associated with ER effectiveness, general arousal, or general valence. The sensory components of pain symptoms (*r* = −0.708, *p* = .023) and health anxiety (*r* = −0.443, *p* = .028) were significantly negatively associated with effective ER.

**Conclusions:**

ER effectiveness seems independent of general somatic symptom distress. We make recommendations for clinical interventions in light of these complex findings.

## INTRODUCTION

What happens when your boss tells you that you are not doing well at your job? William James, Carl Lange, and more recently Antonio Damasio would answer that you feel bodily arousal, which is then communicated to the brain. In healthy people, this then creates the subjective feeling of displeasure, fear, or anger: emotions are seen as a side effect of physiological processes (1,2). However, recent studies point to the fact that this might be different in people with functional somatic symptoms.

Emotional experiences are created by integrating afferent signals from the body (3,4). Changing bodily states can lead to alterations in emotional intensity—for example, manipulating the cardiac baroreflex can lead to heightened fear perception (5). Another recent study found that inducing tachycardia via a pacemaker in mice led to more anxiety-like behavior, but only in risky contexts (6). Although the findings are mixed, some studies found that people who perceive their heartbeat well also experienced emotions more intensely (7,8). The cardiovascular channel seems to be particularly emotionally laden (8).

However, people with chronic somatic symptoms or functional disorders are thought to have difficulties sensing their own bodies (9–11). Somatic symptom disorder (SSD) is characterized by an excessive focus on physical symptom, accompanied by cognitive-affective features such as health anxiety (12).

The current state-of-the-art explanatory model for these disorders represents the predictive processing model (13–18). This model assumes that somatic symptom experience is the result of an active inferential process. This experience is shaped by both prior information (often described as the “hypothesis” a person has about their current bodily state) and the actual (somatosensory) signal, which means that the resulting symptom experience is a posterior in Bayesian terms. Prediction errors resulting from a mismatch between the prior and the actual stimulus (sensory input) are minimized in healthy people. However, in SSDs and related syndromes, these errors are presumably not effectively minimized, with the prior dominating the process (18). The predictive processing model not only does apply to symptom perception but can also be applied to emotions. For example, Van den Bergh’s (19) Better Safe Than Sorry model posits that people with SSDs and related syndromes have strong emotional schemata regarding symptoms (e.g., “every symptom is a threat to my health”), leading to oversimplified threat processing, reduced memory specificity, and compromised fear learning.

Disturbed emotional processing is a hallmark symptom of SSDs—the B criterion in the *Diagnostic and Statistical Manual of Mental Disorders* (Fifth Edition) specifies that the diagnosis can only be given when an excessive cognitive, behavioral, or emotional response to health concerns is present (12). People with persistent somatic symptoms are more likely to show negative affect (20,21). The link between trait negative affect and somatoform disorders is empirically well established (19) and explicitly included in the Hierarchical Taxonomy of Psychopathology (HiTOP) as a novel higher-order superspectrum of emotional dysfunction that combines internalizing and somatoform psychopathology (22).

Failing to *regulate* emotions can have an impact on psychopathology (23). In terms of Gross’s (24) extended process model of emotion regulation (ER), people with SSDs do not only have shortcomings at this situational level, but also in the attention deployment stage and beyond (25). Attention cannot be deployed adequately if an emotion is not perceived effectively—yet people with SSD are more likely to have high trait alexithymia and show difficulties with emotion identification (25–27). If attention is not deployed, one likely cannot change cognitions or adequately modulate a response to an emotional situation. In line with this, people with SSD seem more likely to habitually suppress their emotions and to try to direct their attention away from the situation (28,29). Interestingly, it seems that these patients are not aware that they are suppressing (29). They report to use reappraisal strategies less in daily life and perceive ER strategies within laboratory tasks as more effortful than healthy comparison participants (29). Nevertheless, it remains unclear what the cause of these alterations are and which of these findings are effects of more basal emotional processing steps.

To this end, we designed this study to explore the mechanisms behind ER across people of the general public. We therefore aimed to recruit a broad, heterogeneous sample while slightly oversampling of people with elevated somatic symptom levels. The reason for this design was that symptom reports are a dimensional phenomenon and case-control designs would oversimplify and mask the true underlying variance of this construct (30,31). We hypothesized that somatic symptom distress is significantly positively associated with less functional (e.g., appraisal) and more dysfunctional (e.g., suppression) ER strategies. As an ER paradigm, we used the task by Schnabel and colleagues (29). By applying structural equation model (SEM) analyses with both established and more state-of-the-art, dimensional measures, we aimed at investigating which facets of somatic symptom distress (in the Patient Health Questionnaire-15 [PHQ-15] bifactor model and the somatoform HiTOP model) are particularly related to disordered ER.

## METHODS

### Transparency and Openness

We report how we determined our sample size, all data exclusions, all manipulations, and all measures in the study, and we follow Journal Article Reporting Standards (32). This study’s design and analysis plan are preregistered at osf.io (https://doi.org/10.17605/OSF.IO/43PR9).^1^ Data and code are available at https://doi.org/10.17605/OSF.IO/TDHUV. Ethical approval was granted by the ethical commission at the Department of Psychology of the Johannes Gutenberg-University, Mainz, Germany. Data were collected from February to August 2022.

This project has received funding from the European Union’s Horizon 2020 research and innovation program under the Marie Skłodowska-Curie grant agreement no. 956673. This article reflects only the author’s view; the Agency is not responsible for any use that may be made of the information it contains. The current study is part of the innovative training network ETUDE (Encompassing Training in fUnctional Disorders across Europe; https://etude-itn.eu/), ultimately aiming to improve the understanding of mechanisms, diagnosis, treatment, and stigmatization of functional disorders (33).

### Participants

We aimed for a sample size of *N* ≥ 250, as this is the sample size at which correlation coefficients stabilize (34) and due to requirements of SEM analysis. Exclusion criteria were being below 18 or above 65 years old or having epilepsy or Parkinson’s disease. These conditions were excluded as we were worried about seizures caused by flickering screens or tremor and related motoric symptoms impeding reaction ability, respectively.

Participants were recruited through student mailing lists, press statements, posters in the city center, social media, and multiple social and charitable organizations (such as the Red Cross, the local homeless shelter). We also recruited from lists of potentially interested (former) patients of the university outpatient clinic who had indicated (25) that they were interested in participating in scientific studies and (1) had originally contacted the outpatient clinic with a somatic symptom. The researchers did not receive information on when these persons had first contacted the clinic, whether these persons had completed or even started their treatment, and whether the somatic symptom was the primary reason for their treatment. Because of the sampling procedure, we did not conduct a full psychodiagnostic workup including clinical interviews. Therefore, we do not have exact information regarding current mental disorders.

For their participation, participants were compensated with €30 or study participation credits.

### Materials

Next to the questionnaires described below, we also administered the Patient Health Questionnaire-4 (35), the State Trait Anxiety Inventory–State Inventory Short Form (36), and the Childhood Trauma Questionnaire (37). However, as these were not used for inferential statistics, they are not described below.

#### Patient Health Questionnaire-15

Kroenke (38) also designed the PHQ-15, a 15-item scale that measures somatic symptoms in the last 4 weeks. Responses are given on a three-point scale (0 = *not bothered at all*, 2 = *bothered a lot*). The PHQ-15 has a bifactor structure, consisting of one overarching general factor and several symptom-specific factors (9,39). Again, the German translation and validation is by Löwe et al. (36). A histogram of the distribution in our sample can be seen in Figure S1, Supplemental Digital Content, http://links.lww.com/PSYMED/B18. Here, Cronbach’s *α* = .80.

#### Somatic Symptom Disorder—B Criteria Scale

This questionnaire (40) assesses the B-criterion of the *Diagnostic and Statistical Manual of Mental Disorders* (Fifth Edition) Somatic Symptom Disorder, thereby measuring the thoughts, feelings, and behaviors related to symptoms or health concerns. It spans 12 items with a five-point response scale (0 = never; 4 = very often). We measured an internal consistency of Cronbach’s *α* = .93.

#### Toronto Alexithymia Scale

The Toronto Alexithymia Scale-20 is a 20-item questionnaire measuring alexithymia (41). It consists of three subscales: difficulties identifying feelings, difficulties describing feelings, and externally oriented thinking. The German translation is by Bach et al. (42). Here, Cronbach’s *α* = .79.

#### Somatosensory Amplification Scale

The somatosensory amplification scale reflects how bothersome or worrisome somatic symptoms are experienced (43). It consists of 10 items that are answered on a five-point scale. The internal consistency was Cronbach’s *α* = .80.

#### HiTOP Somatoform Phase 1 Items

This questionnaire, which is based on the HiTOP model (44), was created to find transdiagnostic markers of somatoform and related disorders. It consists of 52 items about bodily experiences in the last 12 months with a four-point scale (45,46). The five subscales are bodily distress symptoms, conversion symptoms, health anxiety, disease conviction, and somatic preoccupation. The German translation is by Hartmann and colleagues (47). Internal consistency was high at Cronbach’s *α* = .93.

#### Emotion Regulation Questionnaire

This questionnaire is a 10-item instrument developed by Gross and John (48). This measure distinguishes between two types of ER: cognitive reappraisal (six items; “I control my emotions by changing the way I think about the situation I’m in”) and expressive suppression (four items; “I keep my emotions to myself.”). Respondents can indicate their answers on a seven-point scale (1 = *strongly disagree*; 7 = *strongly agree*). It has been translated to broad range of languages, adapted to a variety of settings, and shows good psychometric properties (49–52). The German version is by Abler et al. (53). Overall reliability was Cronbach’s *α* = .75.

#### Emotional Reactivity Scale

This questionnaire, originally developed by Nock et al. (54), assesses emotion sensitivity, intensity, and persistence. It consists of 21 items to be rated on a five-point scale (0 = *not at all like me*; 4 = *completely like me*). Three subscales can be identified: sensitivity (8 items, e.g., “My feelings get hurt easily”), arousal/intensity (10 items, e.g., “When I experience emotions, I feel them very strongly/intensely”), and persistence (3 items, e.g., “When something happens that upsets me, it’s all I can think about it for a long time.”). This questionnaire is well validated and consistent (54). Internal consistency was high at Cronbach’s *α* = .94.

#### Emotion Regulation Task

We adapted a task designed by Schnabel and colleagues (29) to assess individual levels of ER abilities, particularly reappraisal and suppression. After the instructions, participants viewed three videos explaining one ER technique each, in random order: reappraisal, suppression, and observation. After each video, participants had 3 trials to practice the respective strategy. There were 48 trials after the practice rounds, which consisted of a negatively valent picture shown for 2.5 seconds, followed by valence and arousal ratings on the self-assessment manikin (Self-Assessment Manikin—these are the prestrategy ratings), then the strategy the participants should use for 3 seconds, followed by the same picture being shown again for 5 seconds. Participants rated valence and arousal on the Self-Assessment Manikin again (poststrategy ratings). After this, they were asked how difficult it felt to apply this strategy on a five-point scale and how well they managed to implement the strategy, also on a five-point scale. This task was presented via Inquisit (55).

### Design and Procedure

All potential participants were screened for exclusion criteria via a telephone call. Participants were asked to complete the questionnaires in their spare time up to 2 days before coming to a lab appointment. In the lab, they were asked to complete the Emotion Regulation Task after providing informed consent.

### Statistical Analysis

The data were analyzed using SPSS version 27 (56), R version 4.1.2 (57), and JASP version 0.16.4 (58). We removed *n* = 39 incomplete data sets (i.e., where only the experimental part [*n* = 11], or only the questionnaires [*n* = 25] were present, or where participants accidentally filled out parts of the questionnaire twice [*n* = 3]), but we did not remove outliers (see (59) for reasons why not to remove outliers). There were no missing data due to each question in the survey and the experimental part needing to be answered. The data were not transformed in any way.

Then, we calculated demographics, frequencies, and reliabilities. We computed mean prestrategy and poststrategy arousal and valence values. Next, we calculated correlations between the measures, both from a frequentist and a Bayesian perspective. These were also used to check the hypothesis Manipulation checks consisted of six paired *t*-tests: three strategies with both an arousal and a valence aspect. Analyses of variance (ANOVAs) were used to do a randomization check and to see differences in pre-post differences in valence and arousal values across strategies.

Lastly, we calculated two SEMs: one with the PHQ-15 and one with the HiTOP-SF. We have shown the appropriateness of the underlying baseline models elsewhere (see (60)). Here, we wanted to examine the connections between symptom burden and ER strategy use in the ER task. For the PHQ-15 model, we were using the single questionnaire items. The weighted least squares with mean- and variance-adjusted chi-square test for goodness-of-testing estimator was used, as it is the best for categorical data with nonequidistant categories, especially in samples with similar sizes to ours (61), and theta parametrization was applied as it performs better in limited information probit modeling approaches (62). We used a similar approach with the HiTOP and its subscales, but because of the large number of model parameters and the resulting computing time, we decided to use subscale scores instead. As these data were continuous, we were able to use a maximum likelihood variance– and mean-adjusted estimator, as this estimation method was shown to be optimal under normality assumptions when there are no missing data (63). The other two models extended these baseline models with parameters from the ER task. We decided to model a bifactor structure consisting of arousal and valence as overarching latent variables on one side, and an ER latent variable considering only reappraisal and suppression on the other side (as observation is not a regulation strategy). Correlations were modeled between the Health Anxiety and Disease Conviction subscales. We further allowed correlations from HiTOP subscales/PHQ-15 sensory factors and all three emotion-related latent variables but did not allow the ER latent variable to correlate with overarching *g*-valence or *g*-arousal. Lastly, strategy-specific valence and arousal were allowed to correlate, but not between strategies.

## RESULTS

### Demographics

In total, *N* = 265 completed the laboratory assessment. *n* = 11 people did not fill in the online questionnaires or had technical difficulties, so the final sample size was *N* = 254 participants. Demographic details can be seen in Table 1.

**TABLE 1:** Demographic Information

	*M* or *n* (%)
Age (range, 18–65), y	28.51 (SD = 10.738)
Women/Men/Nonbinary	174/78/2
Occupation	
Students	189 (74.4%)
Employees	36 (14.2%)
Job-seeking or homemaker	9 (3.6%)
Self-employed	6 (2.4%)
Retired	6 (2.4%)
Civil servants	5 (2.0%)
Other	3 (1.2%)
Education	
Tertiary entrance diploma	138 (54.3%)
University degree	99 (39.0%)
Middle-level high school diploma	9 (3.5%)
Low-level high school diploma	7 (2.8%)
No finished education	1 (0.4%)
BMI*^a^* (range, 15.52–52.06), kg/m^2^	23.43 (SD = 4.815)
Self-reported psychopathological condition	56 (22.0%)
Self-reported depression	38 (15.0%)
Self-reported anxiety	20 (7.9%)
Self-reported somatic condition	52 (20.5%)
Self-reported functional disorder	60 (23.6%)
PMS	21 (8.3%)
IBS	17 (6.7%)
Chronic back pain	17 (6.7%)
Chronic headaches	12 (4.7%)
Tinnitus	11 (4.3%)
Fibromyalgia	4 (1.5%)
Chronic fatigue	2 (0.8%)
On medication	88 (34.6%)

*^a^* Five people declined to state their body weight.

### Hypothesis Check

In the experimental task, symptom severity as measured by the PHQ-15 was significantly negatively associated with the valence effect of reappraisal, but not significantly associated with the arousal effect of reappraisal (*r*_PHQ − 15, valdiff_ = −0.165, *p* < .01, BF_10_ = 2.468; *r*_PHQ −15, arodiff_ = 0.033, *p* = .605, BF_10_ = 0.090). Note that the Bayes factors indicate that evidence for both correlations is inconclusive. The correlations between PHQ-15 on the one hand and valence and arousal effect of suppression on the other hand were also both not significant (*r*_PHQ −15, valdiff_ = 0.028, *p* = .663, BF_10_ = 0.086; *r*_PHQ − 15, arodiff_ = − 0.066, *p* = 296, BF_10_ = 0.135).

Regarding self-report measures, use of reappraisal was significantly negatively associated with higher symptom burden (*r*_PHQ − 15, ERQ_App_ = −0.178, *p* <.001, BF_10_ = 4.468), whereas use of suppression seemed independent of symptom burden (*r*_PHQ − 15, ERQ_Supp_ = − 0.002, *p* = .976, BF_10_ = 0.079).

### Correlations (Frequentist and Bayesian)

The correlations are depicted in Tables S1–S3, Supplemental Digital Content, http://links.lww.com/PSYMED/B18. Emotion Regulation Questionnaire (ERQ) Reappraisal was also significantly negatively correlated to Somatic Symptom Disorder—B Criteria Scale scores (*r*_SSD − 12, ERQ_App_ = 0.271, *p* < .001, BF_10_ = 1108.409), but ERQ Suppression again seemed independent (*r*_SSD − 12, ERQ_Supp__ = 0.027, *p* = .663, BF_10_ = 0.086). Trait emotion regulation measures were not significantly associated with emotion regulation task measures.

### Emotion Regulation Task

As a sign of successful randomization of the pictures, a GLM ANOVA with arousal prestrategy values showed no significant differences (*F*(1.910, 483.196) = 0.977, *p* = .374, *η*^2^ = 0.004, Greenhouse-Geisser corrected for sphericity). The same was the case for prestrategy valence, *F*(2,506) = 1.377, *p* = .253, *η*^2^ = 0.005. This means that participants did not detect differences in the pictures before the strategy to be used on the respective pictures was revealed. For a manipulation check, all six paired *t*-tests were significant at *p* < .001.

The strategy-specific differences in arousal effect (differences between poststrategy and prestrategy) were significant (*F*(1.886, 477.106) = 14.630, *p* < .001, *η*^2^ = 0.055). The largest arousal effect was found in reappraisal conditions (*M*_reapp_ = − 0.370, SE = 0.039), which was significantly different than suppression (*M*_supp_ = − 0.255, SE = 0.037, *p*_reapp vs. supp_ < .001) and observation (*M*_obs_ = − 0.160, SE = 0.035, *p*_reapp vs. obs_ < .001. Observation and suppression were also significantly different from each other (*p* = .023). Valence effect strategy differences were also present when comparing poststrategy and prestrategy timepoints: *F*(1.831, 463.176) = 97.384, *p* < .001, *η*^2^ = 0.278. All three strategies differed from each other at a *p* < .001 level: reappraisal led to the highest valence increase (meaning that after reappraisal, pictures were judged as more pleasant than in other conditions, *M* = 0.911, SE = 0.046), followed by suppression (*M* = 0.500, SE = 0.035), and lastly observation (*M* = 0.358, SE = 0.035). As for effort ratings, reappraisal (*M* = 3.256, SE = 0.041) was seen as more effortful than suppression (*M* = 3.472, SE = 0.044) and observation (*M* = 3.497, SE = 0.043), but there were no differences between the latter (total ANOVA: *F*(1.953; 494.079) = 21.246, *p* < .001, *η*^2^ = 0.078). For adherence, reappraisal had the least adherence (*M* = 3.340, SE = 0.041), followed by observation (*M* = 3.524, SE = 0.043) and suppression (*M* = 3.543, SE = 0.043), but again there were no significant differences between observation and suppression (total ANOVA: *F*(1.874, 474.064) = 16.261, *p* < .001, *η*^2^ = 0.060).

A regression with PHQ-15 score as criterion showed that of all six pre-post differences between valence and arousal per strategy (3 strategies × valence or arousal differences), only the pre-post difference for reappraisal conditions reached significance (*b* = − 1.663, *p* = .018). In other words, being able to regulate arousal by using reappraisal predicted a lower somatic symptom burden.

### Structural Equation Models

**FIGURE 1:**
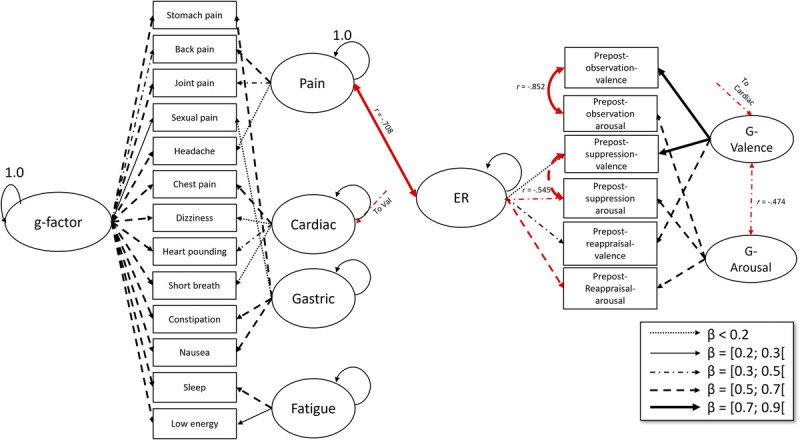
Structural equation model with the bifactor PHQ and emotion regulation strategies. *Note*. Only significant correlations are shown. Double-headed arrows depict correlations, whereas single-headed arrows depict factor loadings of latent variables. Red arrows indicate negative relationships. PHQ = Patient Health Questionnaire. Color image is available online only at the journal website.

Regarding the PHQ-15 bifactor model with ER task, $\chi_{\text{scaled}}^{2}=145,934,\mathrm{df}=120,p=.054,$ fit measures were Comparative Fit Index = 0.980, root mean square error of approximation = 0.029, and standardized root mean square residual = 0.063. As visible in Figure 1 and Table S4, Supplemental Digital Content, http://links.lww.com/PSYMED/B18, the PHQ-15 *g*-factor was not significantly correlated with ER or with *g*-valence or *g*-arousal. The pain sensory variable was significantly and strongly negatively correlated to ER (*r* = 0.708, *p* = .023), indicating an inverse relation between effective ER and sensory pain perceptions. There was a significant negative medium-sized correlation between the cardiac sensory variable and g-valence (*r* = 0.352, *p* = .047), suggesting that cardiac sensory perceptions are associated with less effective ER, but no further correlations between the latent variables.

**FIGURE 2:**
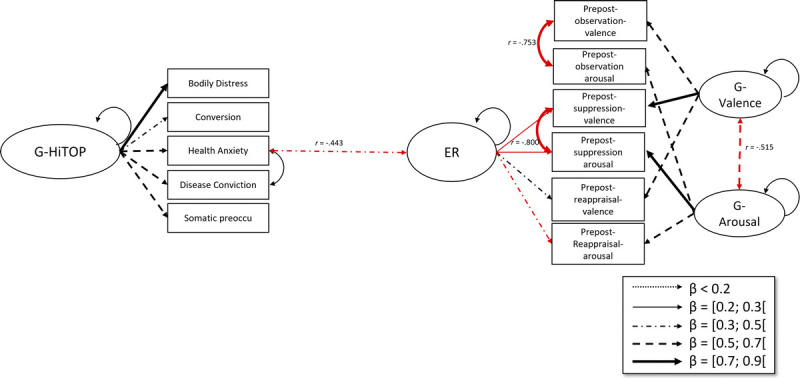
Structural equation model with HiTOP and emotion regulation strategies. *Note*. Only significant correlations are shown. Double-headed arrows depict correlations, whereas single-headed arrows depict factor loadings of latent variables. Red arrows indicate negative relationships. HiTOP = Hierarchical Taxonomy of Psychopathology. Color image is available online only at the journal website.

The HiTOP-and-ER model can be seen in Table S5 (Supplemental Digital Content, http://links.lww.com/PSYMED/B18) and Figure 2. Here, *χ*^2^_scaled_ = 22.903, *df* = 20, *p* = .294. Other fit measures were Comparative Fit Index = 0.996, root mean square error of approximation = 0.027, and standardized root mean square residual = 0.031. The only significant correlation with ER was with the health anxiety residual (*r* = −0.443, *p* = .028), indicating that higher health anxiety is associated with less effective ER. Notice that *g*-valence, *g*-arousal, and ER latent variables were practically independent of the g-HiTOP factor.

## DISCUSSION

In this study, we examined the associations between emotion processing and regulation parameters and facets of somatic symptom distress across a broad and heterogeneous sample. Importantly, facets of symptom distress (as measured by PHQ-15 and HiTOP) were largely uncorrelated with facets of ER. This contrasts the predominant idea that SSDs and related conditions mainly stem from deficits in ER. The findings rather suggest that ER difficulties (at least regarding the facets included in this study) are most likely not a main causal mechanism contributing to somatic symptom distress.

We were only partially able to confirm our hypothesis. The arousal effect of reappraisal was the only significant correlate and the most important predictor of the PHQ-15 score. Valence and arousal after suppression or observation were not correlated to PHQ-15 scores or any other diagnostic marker. Regarding the questionnaire measures, these diagnostic markers were correlated to ERQ-reappraisal, indicating that people with more symptoms use reappraisal less often or effectively in their daily lives, which is in line with findings by Okur Güney et al. (28) and Schnabel et al. (29). It seems that reappraisal might represent a key strategy that researchers should be focusing on when it comes to ER skills across the symptom severity spectrum, which is perhaps related to it being judged as the most effortful and difficult to adhere to. However, all correlations were relatively small, and the findings were limited to the questionnaire data.

The SEM analyses showed no significant correlations between the *g*-factor and the three latent variables of interest (*g*-arousal, *g*-valence, and ER). This suggests that the internal model of attending to symptoms is not the cause of the often-reported ER and processing difficulties in people with SSD. However, the pain sensory factor correlated negatively with ER. Perhaps pain—controlled for motivational factors—represents an unpleasant unspecific underlying sensation, which leads to conservation of mental and physical resources. ER, on the other hand, requires a lot of effort, especially reappraisal, which requires actively engaging with and assessing one’s thoughts and beliefs about unpleasant stimuli. Fittingly, reappraisal valence and arousal contributed more strongly to the ER latent variable than the suppression variables. Lastly, cardiovascular symptoms, as expected (based on (8)), were correlated with overall valence.

As with the PHQ-15, the SEM analysis with the HiTOP-model did not show any significant correlations between the G-SF-HiTOP and the three latent variables. The model showed a significant negative correlation between the health anxiety residual and adaptive ER. Individuals with higher levels of health anxiety often show more rumination (64), which is considered a less adaptive ER strategy and predicts chronicity of negative affect, including depressive symptoms (65). This could mean that these people have difficulties disengaging from these negative thoughts, distracting themselves, or perceiving potential strategies to cope and therefore get “stuck” in the ER process at the selection stage (24). Future studies should investigate whether health anxiety and sensory components of pain have additive or interactive effects, as this is outside of the scope of this paper.

In our study, trait suppression (as measured by the ERQ) and trait reappraisal seem independent. In earlier literature, it was thought that reappraisal is an especially beneficial strategy and that suppression has a negative impact subsequent emotional experiences (see (48), for a full overview). However, newer research suggests a more differential point of view, highlighting the importance of interactions between individual characteristics, the specific situation, and the strategy demands (66). In line with this argument, our research suggests that, although there are benefits of trait reappraisal in terms of diagnostic instruments (e.g., Somatic Symptom Disorder—B Criteria Scale and HiTOP) as well as affective psychopathology (e.g., State Trait Anxiety Inventory, Patient Health Questionnaire-4), there are not necessarily disadvantages for people scoring high on suppression. The only correlation we found with ERQ suppression was alexithymia. This makes sense as the *personal characteristic* being alexithymic and not recognizing emotions means one will have a hard time working through these emotions (=reappraisal) and is more likely to expressively suppress them. Furthermore, there were no correlations between trait and task variables assessing ER. This finding suggests that the *specific situation* of the lab task is a measure of *maximum* performance, whereas the questionnaires are measures of *typical* performance. We recognize that demand characteristics and social desirability may have contributed to the lab task being seen as a maximum effort task, although we minimized this by having experimenters leave the room during this task.

This study shows that there is no easy conclusion to be made about the association between somatic symptoms and ER difficulties—but this also means that not all hope is lost regarding ER skills for people with functional disorders. Instead, people of all somatic symptom severities can do equally well on the ER task. Everyone, regardless of severity, seems to be able to apply these strategies.

A reason for the shortcomings in ER of people with functional disorders that are reported in the literature is probably the difference between performing ER in the lab and performing ER unprompted and spontaneously in daily life. Using adaptive ER strategies like reappraisal might be exerted only when maximum performance is expected, instead of typical performance. In daily life, the problem might lie in recognizing emotional situations and the evoked emotions and encouraging spontaneous use. Perhaps alexithymia prevents people with FD from realizing the benefits of doing so. Helping people with functional disorders recognize emotionally laden situations and their emotions toward these could also improve emotional self-awareness. Instead of overgeneralizing such a situation and experiencing an activation of threat-schemata, people with functional disorders should be encouraged to keep an open mind and thereby update their beliefs/priors toward the situation—see Van den Bergh et al. (19). The person who was just yelled at by their boss and is experiencing unpleasant stomach sensations could, for example, be encouraged to stay focused on the situation and reevaluate their gastrointestinal symptoms in light of the current event. In terms of Gross (24), this could help the person realize they are in the emotional situation and deploy attention to the relevant features of the situation (e.g., the boss is unhappy with my work performance), thus allowing them to change their cognitions and responses to the situation accordingly.

This study is not a randomized controlled trial with a group of people with functional disorders and an equally large control group. Instead, we had one sample in which we oversampled people with elevated somatic symptom levels. Of course, this oversampling could have introduced other potential biases, and the study findings should be replicated in strictly representative samples. Although this design choice may seem like a limitation, this is actually a strength: the purpose of this study was to cross-sectionally investigate how these traits are distributed. Somatic symptom reporting has a dimensional nature and that dichotomizing symptom perception is psychometrically questionable (30,67). When using only two groups, one of which is by definition healthy (because of the usual exclusion criteria) and one is on the psychopathological spectrum, it can be hard to generalize finding to the average human (who usually is somewhere between these two extremes). Mechanisms researchers need to be able to make predictions about a broad range of people with different levels of abilities and comorbidities.

In total, this study finds various correlates of trait- and performance-based ER and provides food for thought on these processes in functional disorders. Our study shows that, although there phenotypically may be some differences in ER abilities between people with and without functional disorders, symptom severity is not the underlying mechanism. Rather, people all across the symptom severity spectrum are able to learn regulation strategies, but some seem to struggle more than others to apply these in their daily lives. More research is needed to obtain a conclusive picture of the mechanisms behind emotional (dys)regulation in functional disorders.

## Footnotes

1 We preregistered another hypothesis in this preregistration: namely, that somatic symptom distress is significantly associated with a less flexible emotion regulation style in light of varying intensities of negative stimulation. Shortly after our preregistration, however, we decided not to include the regulatory choice task because of time constraints. We therefore did not collect data on and cannot (dis)confirm this hypothesis.
